# Epitope-Specific Anti-C1q Autoantibodies in Systemic Lupus Erythematosus

**DOI:** 10.3389/fimmu.2021.761395

**Published:** 2022-01-11

**Authors:** Jessica S. Kleer, Pascal A. Rabatscher, Jessica Weiss, Joel Leonardi, Severin B. Vogt, Andrea Kieninger-Gräfitsch, Carlo Chizzolini, Uyen Huynh-Do, Camillo Ribi, Marten Trendelenburg

**Affiliations:** ^1^ Laboratory of Clinical Immunology, Department of Biomedicine, University of Basel, Basel, Switzerland; ^2^ Division of Internal Medicine, University Hospital, Basel, Switzerland; ^3^ Department of Clinical Pharmacology and Toxicology, University Hospital, Basel, Switzerland; ^4^ Department of Pathology and Immunology, University Hospital, Geneva, Switzerland; ^5^ Department of Nephrology and Hypertension, University Hospital, Bern, Switzerland; ^6^ Division of Immunology and Allergy, Department of Internal Medicine, University Hospital, Lausanne, Switzerland

**Keywords:** autoantibody(ies), autoimmune diseases, complement, systemic lupus erythematosus (SLE), lupus nephritis

## Abstract

**Objective:**

In patients with systemic lupus erythematosus (SLE) complement C1q is frequently targeted by autoantibodies (anti-C1q), that correlate best with active renal disease. Anti-C1q bind to largely unknown epitopes on the collagen-like region (CLR) of this highly functional molecule. Here we aimed at exploring the role of epitope-specific anti-C1q in SLE patients.

**Methods:**

First, 22 sera of SLE patients, healthy controls and anti-C1q positive patients without SLE were screened for anti-C1q epitopes by a PEPperMAP^®^ microarray, expressing CLR of C1q derived peptides with one amino acid (AA) shift in different lengths and conformations. Afterwards, samples of 378 SLE patients and 100 healthy blood donors were analyzed for antibodies against the identified epitopes by peptide-based ELISA. Relationships between peptide-specific autoantibodies and SLE disease manifestations were explored by logistic regression models.

**Results:**

The epitope mapping showed increased IgG binding to three peptides of the C1q A- and three of the C1q B-chain. In subsequent peptide-based ELISAs, SLE sera showed significantly higher binding to two N-terminally located C1q A-chain peptides than controls (p < 0.0001), but not to the other peptides. While anti-C1q were associated with a broad spectrum of disease manifestations, some of the peptide-antibodies were associated with selected disease manifestations, and antibodies against the N-terminal C1q A-chain showed a stronger discrimination between SLE and controls than conventional anti-C1q.

**Conclusion:**

In this large explorative study anti-C1q correlate with SLE overall disease activity. In contrast, peptide-antibodies are associated with specific aspects of the disease suggesting epitope-specific effects of anti-C1q in patients with SLE.

## 1 Introduction

Systemic lupus erythematosus (SLE) is the archetype of a systemic autoimmune disease. It is characterized by a dysregulated immune system, resulting in the generation of autoantibodies to numerous self-antigens and a broad spectrum of clinical manifestations. The exact cellular and molecular mechanisms leading to the disease remain incompletely understood (1) but may be elucidated by exploring the characteristics of self-antigens. One of these self-antigens is C1q, the first component of the classical complement activation pathway. Approximately 20-50% of unselected SLE patients have autoantibodies against C1q (anti-C1q) (2).

Positivity for anti-C1q is predictive for flares of lupus nephritis (LN) and anti-C1q levels correlate with overall disease activity (3). Additional lines of evidence suggest that these antibodies are directly involved in tissue injury (4): C1q deposition is a typical finding in severe LN and anti-C1q could be extracted from glomerular basement membrane fragments (5). Furthermore, C1q is a highly functional molecule (6) and experimental data support the assumption, that binding of anti-C1q alter those functions (7–10). However, the definite pathogenic role of the polyclonal anti-C1q remains to be determined and may strongly depend on the antibody binding site.

C1q is composed of 18 polypeptide chains (6 A-, 6 B- and 6 C-chains), that form six triple helices assembling to a structure that resembles a bouquet of tulips. Each chain has a short N-terminal region, followed by an ~81 residue-long collagen-like region (CLR) forming the stalk of the molecule and a ~135 residue-long C-terminal globular head region (gC1q) (11). The globular heads are mostly responsible for the recognition of target structures, e.g. Fc parts of bound immunoglobulins (12), surface proteins of pathogens and apoptotic cells (13). Upon binding of C1q, the CLR mediates immune effector mechanisms, including complement activation and enhancement of phagocytosis through interaction with cell surface receptors (14, 15).

Anti-C1q are polyclonal and primarily recognize neoepitopes on the CLR of C1q (16, 17) and to a lower extend also on gC1q (18, 19). These epitopes are cryptic, only exposed when C1q is in its bound form (20) and certainly located in different structures. However, so far little is known about the precise C1q epitopes (21, 22). As SLE patient-derived monoclonal anti-C1q Fabs recognize different C1q polypeptide chains in Western blot assay (22), they were used in a previous microarray-based peptide scan to identify peptide sequences recognized by anti-C1q (23). By this approach, Vanhecke et al. described a major linear epitope being located on the N-terminal C1q A-chain covering the arginine rich part of the chain, the so-called ‘A08’. Interestingly, this region is also known as a major binding site for non-immunoglobulin molecules (24), and could even be an early epitope allowing cross reactivity of antibodies that primarily target EBNA-1 of Epstein Bar Virus (EBV) due to sequence homology (25). Epitope spreading might then lead to a more diverse antibody repertoire against the whole C1q molecule. As C1q has more functional subunits than just ‘A08’, e.g. the globular heads, the lysins in the C-terminal CLR that mediate the interaction of C1q with the C1s_2_C1r_2_ tetramer (26) and widely unknown regions, which are responsible for the interaction with C1q receptors (15), antibodies targeting these structures might have different functional consequences and thus mediate different disease manifestations.

The aim of this study was to explore epitopes of C1q and determine whether epitope-specific antibodies against C1q can be linked to specific disease manifestations.

Since the study by Vanhecke et al., was limited to the use of monoclonal antibodies, which do not mirror the polyclonal character of anti-C1q in patients, we used 22 SLE patient sera to determine the epitope landscape of C1q. In addition, we applied an advanced epitope mapping method based on densely overlapping linear as well as cyclized peptides, to increase sensitivity, since many epitopes rely on protein folding, which can hardly be detected with standard microarrays based on linear peptides alone (27). Subsequently we investigated the conspicuous peptide sequences by analyzing a large cohort of well-defined SLE patients provided by the Swiss Systemic Lupus Erythematosus Cohort Study (SSCS).

## 2 Materials and Methods

### 2.1 Blood Samples

#### 2.1.1 Cohort 1

For the epitope mapping, 22 serum or plasma samples were used from healthy blood donors, SLE patients being anti-C1q positive or negative respectively, and anti-C1q positive patients with diseases other than SLE. SLE patients fulfilled at least 4/11 American College of Rheumatology (ACR) revised criteria for the classification of SLE and were all recruited at the University Hospital Basel. Sera from healthy blood donors were obtained from the blood donation center in Basel.

#### 2.1.2 Cohort 2

To determine the association of epitope-specific anti-C1q with disease manifestations, serum samples and related clinical data from 378 SLE patients were provided by the SSCS. SSCS is a prospective, nationwide, multicenter and longitudinal study of SLE patients living in Switzerland (28). SSCS includes adult SLE patients (> 17 years old) who fulfill at least 3/11 ACR revised criteria for the classification as SLE at the time of inclusion and who had given written informed consent. Patients were solely selected based on the availability of complete disease activity scores (SELENA-SLEDAI and PGA) and available plasma sample at the time of study visit.

Plasma samples of 100 healthy, sex matched blood donors from the blood donation center in Basel served as a reference.

### 2.2 Data Collection

Samples and data from SLE patients and healthy blood donors were collected cross-sectionally between October 2010 and June 2018. Laboratory parameters were assessed by the individual centers. SLE manifestations were defined using the ACR revised classification criteria (29, 30). Disease activity was assessed by the Systemic Lupus Erythematosus Disease Activity Index (SLEDAI) score with the Safety of Estrogens in Lupus Erythematosus National Assessment (SELENA) modification (31).

Additionally, we used a Physician’s Global Assessment (PGA) score with a 4-point scale of disease activity, ranging from 0 (inactive) to 3 (very active). Both scores were used with a 30-day window (32). Active SLE disease was defined as a SELENA-SLEDAI ≥ 6 and PGA ≥ 1 at the time of sampling.

### 2.3 Epitope Mapping

Peptide microarrays were manufactured by PEPperPRINT (Heidelberg, Germany). The peptide sequence of the CLR of C1q was laser printed in an array format. Measurements were performed with cyclized peptides of 7, 10, and 13 amino acid (AA) length and with linear peptides of 15 AA length. The cyclic constrained peptides were linked at the C- and N-terminus by a thioether linkage and anchored to the microarray surface. The linear peptides were printed as stripes continually bound to the surface of the microarray. Both linear and conformational cyclic peptides were expressed with a 1-AA shift. Peptide microarrays were screened according to the manufacturer’s protocol (33) with the following specifications: The secondary antibody was a goat anti-human IgG (Fc) DyLight680 and a mouse monoclonal anti-HA (12CA5) DyLight800 antibody was used as a control. Assays were performed with serum or plasma dilutions of 1:500. Arrays were scanned using a LI-COR Odyssey Imaging System and microarray image analyses were done with PepSlide^®^ analyzer. The optical density (OD) was converted to a digital scale leading to values that ranged from 0 to 16,052 arbitrary fluorescence units (aFU) in our study. The magnitude of binding intensity of IgG to certain peptides is presented as color in a Heatmap, in which the highest value was limited to 1’000 aFU to facilitate comparisons between binding intensities of smaller amplitude.

### 2.4 C1q-Derived Peptides

Peptides used for peptide ELISA were synthesized with ≥ 95% purity by peptides & elephants (Hennigsdorf, Germany) and named according to the position of their first AA in the C1q molecule (11). Accordingly, the previously described ‘A08’ (23) was renamed ‘A15’ in this study. The difference in numbering is due to the two AA increments used previously, while the current study used one AA increment being identical with the AA position in the molecule. Peptides used for the experiments are summarized in **Table 1**. The peptides were diluted in Invitrogen™ UltraPuren™ DNase/RNase-Free distilled water and stored at -80°C until further use.

**Table 1:** Nomenclature and structure of the studied peptides.

Previous name	New name	C1qchain	N-term	Sequence
	A09	A	Biotin	GKKGEAGRPGRRGRP
A08	A15	A	Biotin	GRPGRRGRPGLKG
	A86	A	Biotin	NIKDQPRPAFSAIRR
	B41	B	n/a	cyclo[K(biotin)AGDHGEF]
	B43	B	n/a	cyclo[K(biotin)DHGEFGE]
	B83	B	n/a	cyclo[K[biotin)GESGDY]

A15 and A09 both contain the ‘A08’ core sequence (marked gray) described previously (25). The B-chain derived peptides are brought into a cyclic conformation by an amide bond.

### 2.5 Peptide and anti-C1q ELISAs

Peptide and anti-C1q ELISAs were performed as published previously (23), with some modifications to improve the signal to noise ratio. The ELISAs were performed throughout with TBS and the peptide ELISAs were incubated at 33°C instead of 27°C. The incubation step of peptide coating was shortened to one hour. The optimal serum dilutions were found to be 1:50 for the anti-C1q ELISA, as well as for the peptide ELISAs of B-chain derived peptides. The optimal serum dilution for ELISAs of A-chain derived peptides was 1:100. Before diluting the samples to their final concentration, they were vortexed and then centrifuged for 30 minutes at 4°C and 14’000g.

Bound antibodies to peptides or C1q were detected by incubation for 45 minutes (peptides) or one hour (C1q) with alkaline phosphatase (AP)-conjugated goat anti-human Fc(gamma) antibody diluted 1:1’000 (peptide ELISAs) or 1:5’000 (anti-C1q ELISAs). For the peptide ELISAs, the signal obtained from incubating every single sample with diluting buffer instead of peptide was considered background, and this OD value was subtracted from the peptide-specific peptide.

For further analyses we standardized the experiments by expressing the data in units relative to the OD values obtained from a reference SLE serum (set as 1’000 relative Units, reIU), which was used to establish a standard curve. The reference serum showed high level of binding in the peptide ELISA and anti-C1q ELISA respectively and was included on every second plate. Calibration curves were fitted using a sigmoidal four-parameter logistic model. If the background of a peptide ELISA was higher than foreground, reIU were set to zero. If the background-adjusted values were higher than the upper limit of the standard curve, measurements were repeated in a 1:1’000 dilution and if necessary, in a 1:10’000 dilution, for the A-chain derived peptides and in a 1:500, and if necessary 1:5’000 dilution, for the B-chain derived peptides. For each serum, all peptide ELISAs were performed simultaneously.

### 2.6 Statistical Analysis

Statistical analyses and graphical presentations were conducted using R software version 4.0.2. and GraphPad Prism version 9.1.0. Univariate analyses were used to describe baseline characteristics. Data for continuous variables are presented as median with interquartile range (IQR). Categorical data are presented as frequency and percentage.

Non parametric-tests were used throughout, because of a lack of normal distribution in peptide- and anti-C1q ELISA. Correlations were analyzed by Spearman’s correlation coefficient and differences in antibody titers were analyzed by a two‐sided Mann‐Whitney test. Statistical significance was considered as ^*^ *p* ≤ 0.05, ^**^ *p* < 0.01, ^***^ *p* < 0.001, ^****^ *p* < 0.0001 respectively. For the ELISA data, we set a cutoff corresponding to <10% positivity of the controls in all assays. Univariate logistic regression models were used to examine the relationship between positivity in ELISAs and manifestations of SLE, taking the serological measures as predictors and the presence of different disease features as dependent variables. In addition, we examined the association between positivity in peptide ELISAs and disease duration at the time of blood sampling, taking disease activity as a potential confounder into account. Since we performed an explorative study with no prespecified key hypothesis, type I error control was not implemented. Statistical tests are therefore used only for descriptive purposes. We expressed the results of the logistic regression analyses as odds ratios (OR) with associated 95% confidence intervals (95% CI). Intervals have not been adjusted for multiplicity. Subsequently we performed receiver operating characteristic (ROC) curves to compare the diagnostic performance of the peptide- and anti-C1q ELISAs with regard to specific outcomes. To compare the AUC of two ROC curves, DeLong test was used.

### 2.7 Compliance with Ethical Standards

This study was approved by all responsible local ethical committees and Swissethics (Ethical Committee of the Canton Vaud, Switzerland Ref. No. 2017-01434). All procedures performed in this study involving human participants were in accordance with the ethical standards of the research committee and with the 1964 Helsinki declaration and its later amendments or comparable ethical standards. Informed consent was obtained from all individual participants included in the study.

## 3 Results

### 3.1 Patients Characteristics

#### 3.1.1 Cohort 1

Demographic and baseline characteristics of patients used for epitope mapping are summarized in **Supplementary Table 1**. Patient SLE 4 had been described in a previous case report (34). Patients with anti-C1q but disease other than SLE had complement C2 deficiency (n= 2) (35), hypocomplementemic urticarial vasculitis (HUVS) (n= 1) (36) and essential cryoglobulinemia (n= 1) (37).

#### 3.1.2 Cohort 2

A total of 378 patients, of previously selected 392 SLE patients, met the inclusion criteria. The flow diagram of eligible patients is shown in **Supplementary Figure 1**. Demographic and clinical characteristics are summarized in **Table 2**.

**Table 2:** Demographic and clinical characteristics of patients with systemic lupus erythematosus and control group (normal blood donor).

	SLE group, n= 378^†^	Control group, n= 100
Female, n (%)	324 (85.7)	85 (85)
Male, n (%)	54 (14.3)	15 (15)
**Disease Classification at time of inclusion**		
American College of Rheumatology criteria, median (IQR)	5 (4–6)	
**Ethnicity**		
Caucasian, n (%)	280 (74.1)	
African, n (%)	38 (10.1)	
Asian, n (%)	37 (9.8)	
Native American, n (%)	18 (4.8)	
Other, n (%)	2 (0.5)	
Unknown (%)	3 (0.8)	
**Age**		
At blood sampling, median (IQR)	42 (32–54)	48 (38–60)
Disease duration since Diagnosis of SLE (IQR)	5 (1–13)	
**Disease Activity and Clinical Features**		
Active disease ‡, n (%)	131 (34.7)	
Fever, n (%)	24/377 (6.4)	
Arthritis, n (%)	84/375 (22.4)	
Active muco-cutaneous involvement §, n (%)	119/373 (31.9)	
Vasculitis, n (%)	8/377 (2.1)	
Serositis, n (%)	22/372 (5.9)	
CNS involvement ¶, n (%)	12/375 (3.2)	
Leukopenia, n (%)	53/372 (14.2)	
Thrombocytopenia, n (%)	31/373 (8.3)	
Proteinuria, n (%)	56/298 (18.8)	
Hematuria, n (%)	63/340 (18.5)	
Low Complement, n (%)	112/341 (32.8)	
Anti-ds-DNA antibodies, n (%)	167/340 (49.1)	
Anemia, n (%)	126/371(34.0)	
Elevated ESR #, n (%)	103/339 (30.4)	
Anti-Phospholipid antibodies, n (%)	59/183 (32.2)	

^†^n=378 unless otherwise stated, ^‡^active disease was defined as SELENA- SLEDAI ≥ 6 and PGA ≥ 1, §Active muco-cutaneous involvement defined as malar rash or mucosal ulcers or alopecia at time point of blood sampling, ¶CNS involvement was defined as psychosis, seizure or organic brain syndrome at time of blood sampling, ^#^ESR= erythrocyte sedimentation rate.

Of the 378 patients 324 (85.7%) were female and 54 (14.3%) were male. The median age at the time of blood sampling was 42 (32–54) years and the median SLE disease duration since diagnosis was 5 (1–13) years. At the time of the study visit 131/378 (34.7%) of the patients had active disease, defined as a PGA ≥ 1 and SELENA-SLEDAI ≥ 6 (31). The main clinical manifestations of the study population as defined by the SELENA-SLEDAI are shown in **Table 2**. The sex-matched control group consisted of 85 (85%) women and 15 (15%) men. Their median age at the time of blood sampling was 48 (38–60) years.

### 3.2 Epitope Mapping

In a first step we investigated the binding of IgG to peptides covering the CLR of the C1q A-, B- and C-chain, using peptides in different lengths and in a linear as well in a cyclic conformation. We initially tested 8 samples; four from anti-C1q positive SLE patients, two from anti-C1q negative SLE patients and two from healthy control donors.

**Figure 1:**
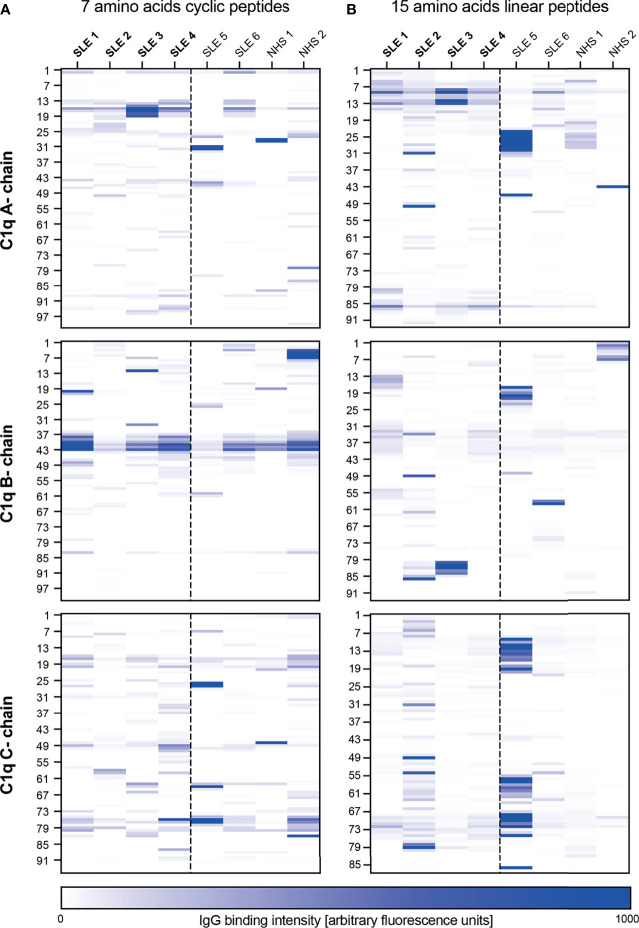
Epitope mapping of the collagen-like region of C1q. Six patients with SLE and two healthy blood donors were screened for antibodies against peptides of the CLR of C1q (A-, B- and C-chain). The heatmap color represents the intensity of the antibody binding signal in each sample (column) to each peptide, named according to the position of their first AA on the C1q molecule (rows, left site). Patients in bold were anti-C1q positive at the time of blood collection, all others anti-C1q negative. **(A)** 7 AA peptides in cyclic confirmation. **(B)** 15 AA linear peptides.

Results are shown in **Figure 1**. Signal intensities from 10 and 13 AA cyclic peptides yielded similar results independent of the peptide lengths and were less strong than from 7 AA peptides. Considering the 7 AA cyclic peptides, a peak-signal was observed in peptides 13 to 19 of the A-chain, all containing the previously described ‘A08’ core sequence (25). The cyclic B-chain derived peptides showed two peaks at position 41 and 43, which were not present in the linear conformation. In the following these peptides are called B41 and B43. The C-chain derived peptides did not show consistent signal elevations. Within the 15 AA peptides in linear conformation, two constant signals appeared at position 9 and 86 of the C1q A-chain, hereafter referred to as A09 and A86 respectively. With regard to the B- and C-chains we did not observe patterns of binding intensities shared by several SLE patients to any of the 15-AA peptides in linear conformation.

In a second step we analyzed 16 additional serum samples, covering a broader disease spectrum, but limited the analysis to 7 AA cyclic peptides from the whole CLR of the C1q B-chain and the N-terminal part of the C1q A-chain covering the already described ‘A08’ epitope. Among all samples, 2 sera were investigated in both experiments and served as an internal control. Results are shown in **Supplementary Figure 2**. Binding intensities to peptides B41 and B43 were detected in all patient groups, but were slightly lower in healthy donors. Similarly, binding to peptide position 83 of the B-chain (B83) can be seen in all patient groups, although less pronounced in the control group.

### 3.3 Prevalence and Clinical Association of Autoantibodies

**Figure 2:**
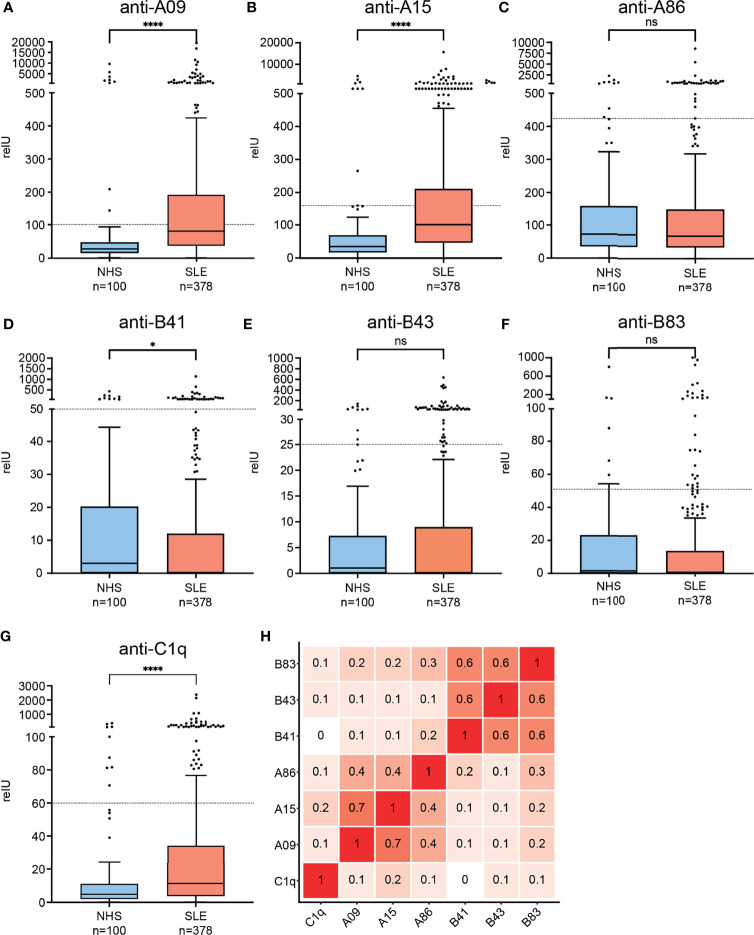
Binding of IgG from SLE patients and healthy controls to candidate epitopes of the collagen-like region and correlation of autoantibodies among each other. **(A–G)** Graphs are named according to examined epitopes and show Tukey’s boxplots with whisker lengths of 1.5x interquartile range. Outliers are shown as dots. Since the data are markedly skewed, Y-axis is segmented. Cutoffs for positivity are indicated by dashed lines. Statistical significance was considered as *p ≤ 0.05, ****p < 0.0001 respectively, ns, not significant. **(H)** Correlation-plot showing spearman correlation coefficients of measured autoantibodies among each other.

To characterize the clinical significance of the candidate epitopes which stand out in the epitope mapping, we established a peptide ELISA. Examined peptides are shown in **Table 1**. As an internal control, we measured antibodies against A15 (anti-A15; formerly called ‘anti-A08’) and anti-C1q as well. **Figure 2** shows the distribution of measured autoantibodies in SLE patients and controls as well as their correlation among each other. SLE IgG showed significantly higher binding to C1q, A15 and A09 and slightly lower binding to B41 when compared to controls. No significant differences in IgG binding was observed for the other peptides.

Since anti-C1q autoantibodies are present in up to 10% of healthy individuals and in analogy to previous study data (38, 39), we chose a cutoff for positivity by accepting <10% positive healthy blood donors in all assays. With this cutoff, 65/378 (17%) of the SLE patients were anti-C1q positive, 159/378 (42%) anti-A09 positive, 123/378 (32.5%) anti-A15 positive, 34/378 (8.9%) anti-A86 positive, 32/378 (8.5%) anti-B41 positive, 53/378 (14%) anti-B43 positive and 29/378 (7.7%) anti-B83 positive. Antibodies directed against C1q-derived epitopes correlated weakly with antibodies against intact C1q (anti-C1q) (r= 0.1 - 0.2). Anti-A09 and anti-A15 showed a strong correlation with each other (r= 0.7), but both had only a moderate correlation with anti-A86 (r= 0.4). Auto-antibodies directed against B-chain derived epitopes showed strong correlations among each other (r= 0.6) but only weak correlations to A-chain derived epitopes (r= 0.1 – 0.3).

**Figure 3:**
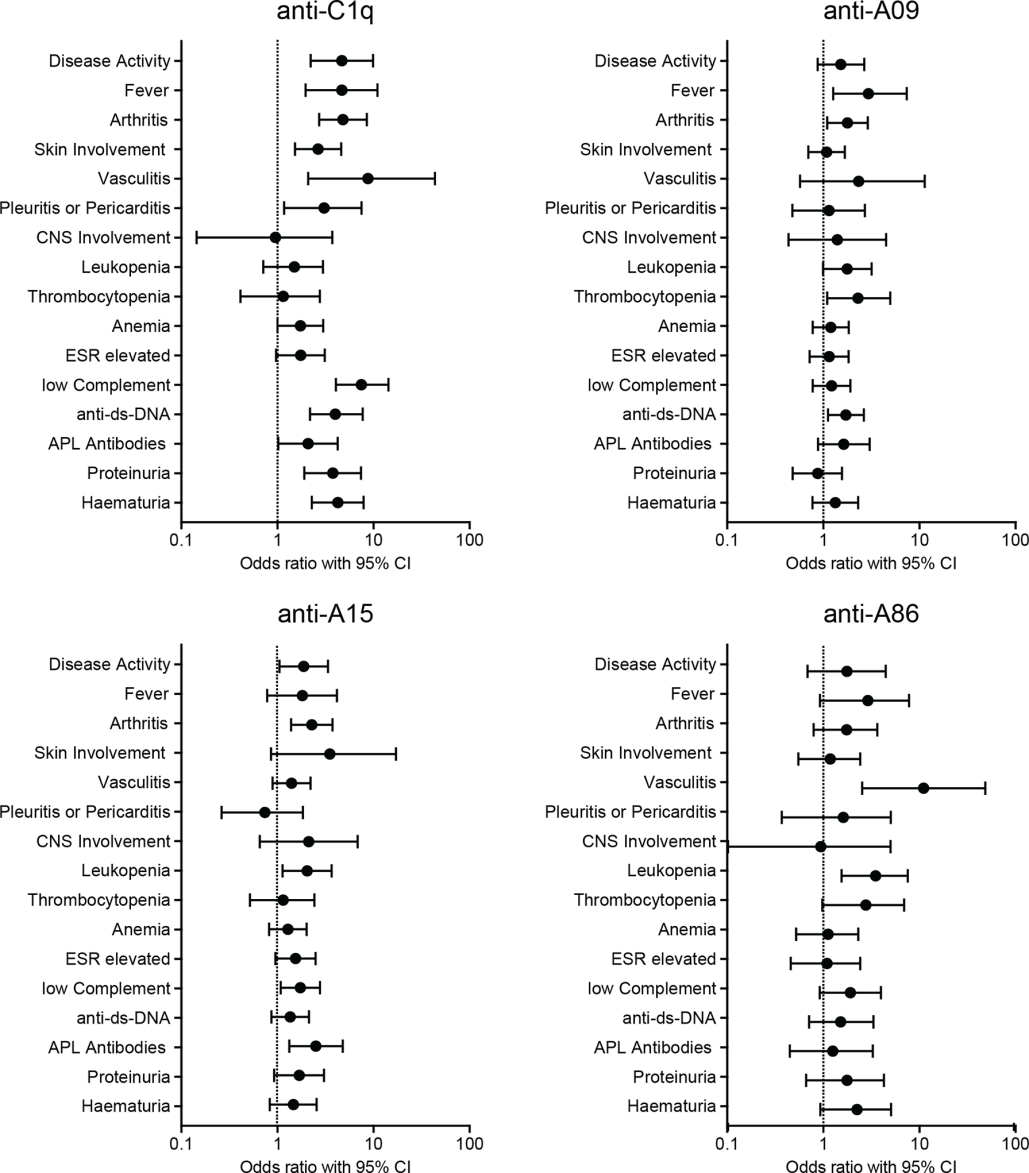
Univariate Logistic Regression. Positivity in ELISAs as binary predictor and presence of disease manifestations as binary dependent variable. The graphs show odds-ratios and 95% confidence intervals of SLE manifestations. ESR, erythrocyte sedimentation rate; APL, antiphospholipid.

To explore the assumed relationship between measured autoantibodies and manifestations of SLE, univariate logistic regression was conducted, taking positivity in ELISAs as binary predictor and the presence of different disease features as binary dependent variable. **Figure 3** shows OR’s and 95% CI’s of SLE features as a function of positivity in anti-C1q- and A-chain derived peptide ELISAs. The corresponding values in numbers are shown in **Supplementary Table 2**.

While anti-C1q positivity correlated strongly with overall disease activity as well as with several SLE features, positivity for autoantibodies to A-chain derived peptides correlated only weakly with disease manifestations: Anti-A09 correlated with fever, arthritis, thrombocytopenia, and with the occurrence of anti-dsDNA antibodies. Patients with anti-A15 had an increased probability of having active disease, arthritis, leukopenia, low complement and antiphospholipid antibodies. Patients with anti-A86 were more likely to have vasculitis and leucopenia. Univariate logistic regression for anti-B-chain derived peptide-ELISAs showed solely a weak correlation between anti-B43 and arthritis (OR= 1.995, CI= 1.046 - 3.708) and anti-dsDNA antibodies (OR= 2.387, CI= 1.232 - 4.824) and are shown in **Supplementary Figure 3** and **Supplementary Table 3**.

**Figure 4:**
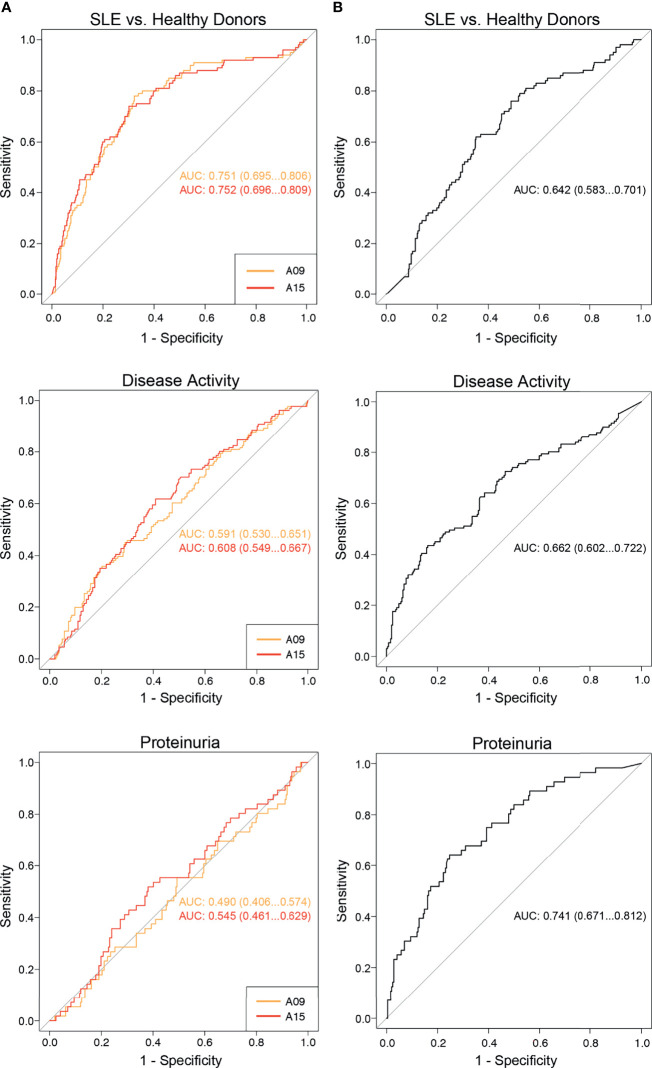
Comparison of the diagnostic performance between anti-C1q and anti-A09/A15 as determined by ELISA. ROC curves analyzing the diagnostic performance of anti-A09, anti-A15 and anti-C1q regarding the discrimination of SLE patients from healthy donors, SLE patients with active versus inactive disease and proteinuria versus no proteinuria. **(A)** ROC curves of anti-A09 and anti-A15. **(B)** ROC curves of anti-C1q.

Since it was previously described that anti-A15 (formerly called ‘anti-A08’) show a stronger correlation with SLE disease activity and nephritis than anti-C1q (10), we established additional ROC curves to allow a better interpretation of the diagnostic performance regarding those endpoints. ROC curves are shown in **Figure 4**. Regarding the discrimination between SLE patients and healthy donors, autoantibodies directed against the N-terminal part of the A-chain had a significantly larger AUC than anti-C1q (0.75 versus 0.64; p < 0.01). In contrast, the diagnostic performance of those autoantibodies taking the outcome active disease into account was not significantly different (AUC anti-A09: 0.59, AUC anti-A15: 0.61, AUC anti-C1q: 0.66; p= 0.09 for comparison of anti-A09 vs anti-C1q and p= 0.19 for anti-A15 vs anti-C1q). Regarding the occurrence of proteinuria, which is a typical finding in LN, anti-C1q show significantly better diagnostic performance than anti-A09 and anti-A15 respectively (AUC anti-A09: 0.49, AUC anti-A15: 0.55, AUC anti-C1q: 0.74; p < 0.0001 comparing anti-C1q versus anti-A09 and anti-A15 respectively).

To investigate the association between anti-C1q, -A09 and -A15 and disease duration we conducted a multivariate logistic regression, adjusting for disease activity. **Supplementary Figure 4** shows the graphical presentation of this multivariate regression using disease duration and activity as predictors and the possibility of positive autoantibodies as outcome. Adjusted Odds ratio for being autoantibody positive per one year of disease duration was 0.94 (CI’s= 0.9 – 0.98) for anti-C1q, 0.98 (CI’s= 0.95 - 1) for anti-A09 and 0.97 (CI’s= 0.95 – 0.99) for anti-A15. The probability of having active disease was markedly higher when having positive anti-C1q (OR_adj_= 4.86, CI’s= 2.67- 9.08), than anti-A09 (OR_adj_= 1.43, CI’s= 0.9 - 2.27) or anti-A15 antibodies (OR_adj_= 1.67, CI’s= 1.03 – 2.68). There was no evidence of multicollinearity.

## 4 Discussion

This study aimed at identifying and exploring the clinical relevance of epitope specific autoantibodies against complement C1q (anti-C1q) in patients with SLE. Considering the multiple functions of C1q, the role of C1q in SLE as well as the striking association of anti-C1q with active LN and SLE disease activity, a better understanding of epitopes of C1q could aid understanding the pathogenic mechanisms of SLE as well as improving diagnostic procedures. By using SLE patient sera in an advanced epitope mapping method, we first identified three epitopes on the C1q A- and three on the B-chain. In subsequent exploration of clinical relevance of these epitopes in a large cohort of patients by peptide-ELISA, two of the investigated peptides were significantly better recognized by serum IgG of SLE patients than of healthy controls. In addition, positivity for four of the investigated peptide-specific antibodies showed associations with selected SLE disease manifestations. These primarily explorative analyses might point to distinct functional properties of the measured peptide-specific antibodies.

The most obvious association with SLE was found for IgG antibodies targeting an epitope on the N-terminal C1q A-chain. The corresponding peptides were named ‘A09’ and ‘A15’ respectively, based on the position of their first AA on the CLR of C1q. Anti-A09 correlated with fever, arthritis, thrombocytopenia, and the occurrence of anti-dsDNA antibodies, while patients with anti-A15 had an increased probability of having active disease, arthritis, leukopenia, low complement and antiphospholipid antibodies. Regarding the occurrence of proteinuria, which is a typical finding in lupus nephritis, anti-C1q showed significantly better diagnostic performance than anti-A09 as well as anti-A15. This finding apparently is not in line with previous studies which showed that anti-A15-ELISA is more specific and more sensitive than a conventional anti-C1q assay for the detection of active lupus nephritis in SLE patients (10) (23). These differences in observation might be explained by the differences in patient selection and number. Both previous studies examined exclusively (10) or predominantly (23) lupus patients with renal biopsy-proven lupus nephritis, whereas most patients analyzed in the present study had long lasting, stable disease resembling an unselected clinical outpatient cohort of patients with closely monitored disease. Furthermore the sample sizes of the preceding studies were substantially smaller, than in the present study, namely n=61 (23) and n= 210 (10) versus n= 378 in our study presented here.

Nevertheless the present study is in line with the study from Vanhecke et al. showing that anti-A15 is better in discriminating asymptomatic donor sera from SLE patient sera than anti-C1q. In the present study, this discrimination was in the same range as the reported diagnostic performance of anti-dsDNA antibodies (40). Hence, anti-A-15 might serve as a diagnostic marker for SLE. However, to determine the real discriminatory power, it will be of importance to also investigate anti-A09 and -A15 in other inflammatory rheumatic diseases, and to perform a direct comparison with anti-dsDNA antibodies.

Furthermore, anti-C1q showed a weak correlation with anti-A09 or anti-A15 antibodies, respectively as also observed by Vanhecke et al. (23). In line with these findings, Wu et al. recently described that anti-A15 antibodies derived from 10 lupus nephritis patients bound to A15 but not to intact C1q (41). Regarding the potential functional consequences of anti-A09 and -A15, it should be noted that both peptides include a major binding site of C1q for non-immunoglobulin molecules (24). With regard to the interaction of A09 compared to A15 with binding partners other than anti-C1q, we hypothesized that the arginines being present in A09 as well will lead to very similar interactions as observed for A15. However, the differences in correlation with clinical parameters between anti-A09 and ant-A15 suggest that either the antibodies have a different potential to interfere with the known interactions of C1q with the described non-immunological molecules and/or receptors, or may point to differences in interaction between the two sites themselves with these binding partners.

The mentioned core sequence was previously described to allow crossreactivity between antibodies directed against EBNA-1 of EBV and C1q (25). In addition, Wu et al. could show that BALB/C mice, which were immunized with the A15 peptide, developed anti-C1q antibodies. They concluded that A15 is important for development of anti- C1q antibodies, but epitope spreading might then lead to a more diverse antibody repertoire against the whole C1q molecule. In line with this finding, generation of anti-A09 and -A15 in SLE patients seem to be an early event in the course of the disease. Additionally, data from multivariate regression suggest that anti-A09 and -A15 have higher stability over time with lower dependency on disease activity (**Supplementary Figure 4**). Taken together, these results support the hypothesis, that molecular mimicry is an early event in the pathogenesis of SLE, with the formation of anti-A15 antibodies being an intermediate step, but might also explain the weak correlation between anti-A15 and anti-C1q.

With regard to IgG antibodies against the other described peptide epitopes, no overall differences in antibody levels between SLE patients and control sera were observed. However, when judging on the significance of these antibodies, one has to keep in mind that quantitatively peptide-specific anti-C1q are only representing a small fraction of total anti-C1q, and they only occur in a subgroup of patients that is likely to be too small to have an impact on overall differences between unselected SLE patients and healthy controls. As the study hypothesis was that antibodies against distinct epitopes of the multifunctional C1q molecule are associated with a specific disease expression, we thus also explored the association of the peptide antibody positivity with the clinical presentation of SLE. Patients with anti-A86 were more likely to have vasculitis and leucopenia, and the presence of anti-B43 was associated with arthritis and anti-dsDNA antibodies.

However, because of the explorative character of these analyses, confirmatory studies in large cohorts of SLE patients covering a broad spectrum of SLE manifestations and taking interrelations into account will be required to define the definite role of the described peptide antibodies.

Lastly, with regard to anti-C1q levels we observed that anti-C1q are associated with a much wider range of clinical disease manifestations than previously described. So far, anti-C1q antibodies have mainly been studied in association with LN (42–44). However, while confirming this known association in the present study, we also observed a clear association with arthritis OR= 4.811 (2.722 - 8. 543), skin involvement OR= 2.646 (1.522 -4.613), vasculitis OR= 8.757 (2.093 -43.629) and serositis OR= 3.065 (1.175 - 7.514). Therefore, anti-C1q could be more broadly considered as marker of SLE disease activity. This observation could be due to the large number of SLE patients investigated in our study. To the best of our knowledge, to date our study is the largest ever on anti-C1q in SLE patients.

The main limitation of the present study is its retrospective observational character. In addition, in spite of the large number of investigated patients, the sample size was still too small to make a clear statistical statement for some of the investigated disease features and thus would require even larger cohorts. Moreover, longitudinal data on the described antibodies will be of importance in the future. Lastly, despite the extensive character of our epitope mapping, the expression of peptides only partially resembles the conformation of the corresponding peptide sequences as part of the complete C1q molecule, and the expression of peptides probably differed between their expression in the initial epitope mapping versus the ELISAs performed in the large SLE cohort. Furthermore, our methodologies were not able to detect and describe antibodies against epitopes involving two or more chains of C1q. Thus, the described peptide-specific antibodies are likely still representing only a fraction of total anti-C1q.

In conclusion, in this exploratory and largest study to date on anti-C1q in SLE patients we describe six candidate epitopes of anti-C1q and their clinical associations in SLE patients. Two N-terminal located A-chain epitopes, which provide good discrimination between SLE patients and healthy individuals, might serve as a biomarker of the disease. In addition, peptide-specific anti-C1q were found to be associated with specific disease manifestations, but their potential impact on clinical patient management and for the understanding of pathogenic mechanisms needs to be confirmed.

## Data Availability Statement

The original contributions presented in the study are included in the article/**Supplementary Material**. Further inquiries can be directed to the corresponding author.

## Ethics Statement

The studies involving human participants were reviewed and approved by Swissethics (ethical committee of the Canton Vaud, Switzerland Ref. No. 2017-01434). The patients/participants provided their written informed consent to participate in this study.

## Author Contributions

All authors were involved in drafting the article or revising it critically for important intellectual content, and all authors approved the final version to be published. JK had full access to all of the data in the study and takes responsibility for the integrity of the data and the accuracy of the data analysis. Study conception and design. JK, JW, MT. Acquisition of data. JK, JL, AK-G, CC, UH-D, CR, and MT. Analysis and interpretation of data. JK, PR, SV, and MT.

## Funding

This work was supported by a project grant of the Swiss National Science Foundation (SNSF) given to MT (320030_200423).

## Conflict of Interest

The authors declare that the research was conducted in the absence of any commercial or financial relationships that could be construed as a potential conflict of interest.

## Publisher’s Note

All claims expressed in this article are solely those of the authors and do not necessarily represent those of their affiliated organizations, or those of the publisher, the editors and the reviewers. Any product that may be evaluated in this article, or claim that may be made by its manufacturer, is not guaranteed or endorsed by the publisher.
