# Supplementary material for: Epitope-Specific Anti-C1q Autoantibodies in Systemic Lupus Erythematosus
Source: Front Immunol. 2022 Jan 11;12:761395. doi: 10.3389/fimmu.2021.761395 (PMC8788646; doi:10.3389/fimmu.2021.761395)
Supplement: Supplementary file 1 [file DataSheet_1.docx]

***Supplementary Material***

Supplementary Table 1. Baseline demographics and disease characteristics in patients used for epitope mapping

| Sample | Dx. | Anti-C1q IgG | Clinical state | SLEDAI score | Age [y] | sex | Class of Lupus Nephritis (WHO) | Disease-Duration [y] |
| --- | --- | --- | --- | --- | --- | --- | --- | --- |
| SLE 1 | SLE | pos. | active | 16 | 54 | m | IV | 0 |
| SLE 2 | SLE | pos. | active | 17 | 52 | f | IV | 1,5 |
| SLE 3 | SLE | pos | active | 10 | 24 | f | III | 0 |
| SLE 4 | SLE | pos. | active | 10 | 32 | f | IV | 0 |
| SLE 5 | SLE | neg. | inactive | 0 | 76 | m | III | 11 |
| SLE 6 | SLE | neg. | active | 42 | 70 | f | - | 0 |
| SLE 7 | SLE | pos. | moderate | 6 | 52 | f | - | 21 |
| SLE 8 | SLE | pos. | active | 24 | 44 | f | IV | 4 |
| SLE 9 | SLE | pos. | active | 38 | 49 | m | IV | 12 |
| SLE 10 | SLE | neg | inactive | 6 | 65 | f | III | 13 |
| SLE 11 | SLE | neg. | inactive | 3 | 62 | f | IV | 26 |
| SLE 12 | SLE | neg. | inactive | 5 | 50 | f | V | 18 |
| C2d1 | other | pos. | active | - | 57 | m | - | 9 |
| C2d2 | other | pos. | inactive | - | 58 | f | - | - |
| HUVS | other | pos. | active | - | 59 | f | IIa, Vb | unk. |
| Cryo | other | pos. | active | - | 52 | m | secondary  MPGN type I | 4 |
| NHS 1 | - | neg. | - | - | 31 | f | - | - |
| NHS 2 | - | neg. | - | - | 33 | f | - | - |
| NHS 3 | - | neg. | - | - | 34 | m | - | - |
| NHS 4 | - | neg | - | - | unk. | unk. | - | - |
| NHS 5 | - | neg. | - | - | unk. | unk. | - | - |
| NHS 6 | - | pos. | - | - | 28 | f | - | - |

Characteristics of Patients included in the epitope-mapping. The clinical state was classified according to physician’s global assessment index at the time of study visit. Anti-C1q-status was determined in the University Hospital Basel and by non-commercial anti-C1q ELISA. SLE = Systemic Lupus Erythematosus, NHS = Normal Human Serum, C2d1 and C2d2 = patients with complement C2 deficiency, HUVS = Hypocomplementic Urticaria Vasculitis Syndrome, Cryo = Patient with severe primary cryoglobulinemia, Dx= Diagnosis, SLEDAI = Systemic Lupus Erythematosus Disease Activity Index, ACR = American College of Rheumatology * assessment at time of sampling

Supplementary Figure 1. Flow chart of included and excluded patients.

Supplementary Figure 2. Epitope mapping of the CLR of C1q. Among the 16 samples used for the second epitope mapping, 2 were investigated in both experiments and served as controls (SLE 4 and 6). The appendix (a) refers to results obtained in experiment 1 and (b) for results obtained in experiment 2. Experiment 2 was limited to 7 amino acid cyclic peptides, derived from the whole CLR of the C1q B-chain and the N-terminal part of the C1q A-chain covering the ‘A08’ epitope (renamed A15). The heatmap color represents the intensity of the antibody binding in each sample (column) to each peptide, named according to the position of their first amino acid on the C1q molecule (rows, left). Patients in bold were anti-C1q positive at the time of blood collection, all others anti-C1q negative. (A) results of the binding intensities to peptides comparing binding intensities measured in the first versus the second experiment. (B) Pooled analyses of IgG binding to peptides in different patient groups.


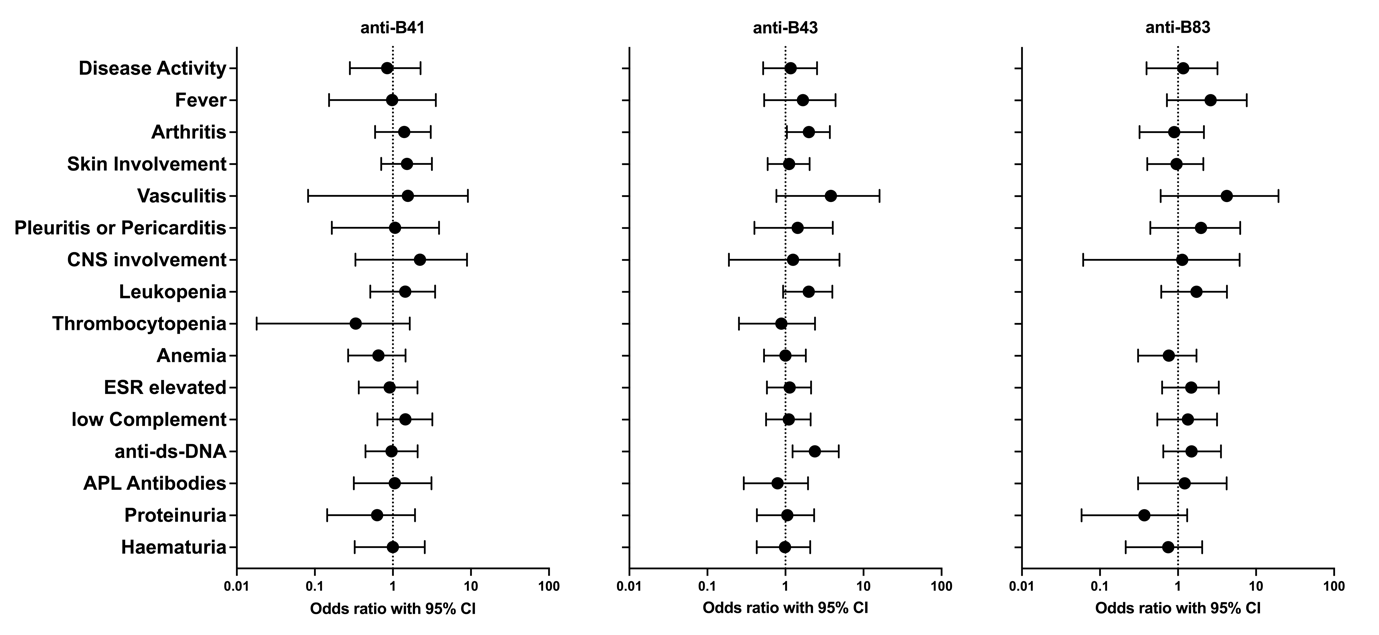


Supplementary Figure 3. Univariate logistic regression with positivity in ELISAs as binary predictor and presence of disease features as binary dependent variable. The graphs show odds-ratios and 95% confidence intervals of SLE features. ESR= erythrocyte sedimentation rate. APL= antiphospholipid-antibodies. Odds ratio for thrombocytopenia in anti-B83 positive patients could not be calculated, since no anti-B83 positive patient had thrombocytopenia.

Supplementary Table 2. ORs and corresponding 95% CI’s resulting from univariate logistic regression, using anti-C1q, anti-A09, anti-A15 and anti-A86 as predictor

| Disease Feature | anti-C1q | anti-A09 | anti-A15 | anti-A86 |
| --- | --- | --- | --- | --- |
| Disease Activity | 4.596 (2.22 - 9.889) | 1.528 (0.871 - 2.688) | 1.905 (1.06 - 3.427) | 1.77 (0.683 - 4.525) |
| Fever | 4.685 (1.963 -11.026) | 2.972 (1.272 - 7.501) | 1.845 (0.787 - 4.253) | 2.94 (0.922 - 7.95) |
| Arthritis | 4.811 (2.722 - 8.543) | 1.791 (1.1 - 2.931) | 2.317 (1.406 - 3.82) | 1.756 (0.791 - 3.695) |
| Skin Involvement | 2.646 (1.522 -4.613) | 1.084 (0.697 - 1.681) | 1.424 (0.899 - 2.248) | 1.183 (0.548 - 2.44) |
| Vasculitis | 8.757 (2.093 -43.629) | 2.353 (0.569 - 11.609) | 3.95 (0.866 – 17.738) | 11.3 (2.559 – 50.014) |
| Pleuritis or Pericarditis | 3.065 (1.175 - 7.514) | 1.151 (0.474 - 2.738) | 0.747 (0.262 - 1.869) | 1.625 (0.367 - 5.112) |
| CNS involvement | 0.952 (0.144 - 3.725) | 1.404 (0.432 - 4.568) | 2.157 (0.661 -7.032) | 0.94 (0.051 - 5.073) |
| Leukopenia | 1.503 (0.714 - 2.979) | 1.78 (0.993 - 3.217) | 2.074 (1.145 - 3.745) | 3.536 (1.554 - 7.69) |
| Thrombocytopenia | 1.151 (0.413 - 2.762) | 2.312 (1.1 -5.044) | 1.16 (0.52 - 2.464) | 2.8 (0.974 - 7.05) |
| Anemia | 1.736 (1.002 - 2.991) | 1.198 (0.776 - 1.848) | 1.303 (0.826 - 2.048) | 1.123 (0.519 - 2.33) |
| ESR† elevated | 1.745 (0.968 - 3.108) | 1.152 (0.719 -1.838) | 1.568 (0.964 - 2.541) | 1.094 (0.456 - 2.445) |
| low Complement | 7.482 (4.072 - 14.297) | 1.222 (0.775 - 1.927) | 1.759 (1.094 - 2.824) | 1.928 (0.916 - 4.028) |
| anti-ds-DNA | 4.004 (2.181 - 7.733) | 1.724 (1.12 - 2.664) | 1.377 (0.875 - 2.172) | 1.521 (0.708 - 3.364) |
| APL‡ - Antibodies | 2.086 (1.021 - 4.246) | 1.639 (0.879 - 3.073) | 2.559 (1.345 -4.906) | 1.256 (0.445 - 3.312) |
| Proteinuria | 3.772 (1.904 - 7.4) | 0.873 (0.477- 1.572) | 1.713 (0.932 - 3.112) | 1.778 (0.661 - 4.328) |
| Hematuria | 4.255 (2.28 - 7.911) | 1.339 (0.771 - 2.322) | 1.486 (0.837 - 2.606) | 2.263 (0.931 - 5.152) |

ESR = erythrocyte sedimentation rate. APL = antiphospholipid. Odds ratio for thrombocytopenia in anti-B83 positive patients could not be calculated since no anti-B83 positive patient had thrombocytopenia.

Supplementary Table 3. ORs and corresponding CI’s resulting from univariate logistic regression, using anti-B41, anti-B43 and anti-B83 as predictor

| Disease Feature | anti-B41 | anti-B43 | anti-B83 |
| --- | --- | --- | --- |
| Disease Activity | 0.845 (0.281 - 2.267) | 1.168 (0.517 - 2.544) | 1.166 (0.395 - 3.2) |
| Fever | 0.979 (0.152 - 3.549) | 1.672 (0.535 - 4.387) | 2.624 (0.722 - 7.602) |
| Arthritis | 1.398 (0.591- 3.057) | 1.995 (1.046 - 3.708) | 0.896 (0.322 - 2.15) |
| Skin Involvement | 1.517 (0.708- 3.161) | 1.115 (0.592 - 2.041) | 0.957 (0.403 - 2.113) |
| Vasculitis | 1.558 (0.082 - 9.159) | 3.828 (0.766 - 16.094) | 4.222 (0.599 - 19.365) |
| Pleuritis or Pericarditis | 1.067 (0.165 - 3.901) | 1.433 (0.401 - 4.042) | 1.968 (0.442 - 6.267) |
| CNS involvement | 2.22 (0.331 - 8.924) | 1.252 (0.189 - 4.928) | 1.131 (0.061 - 6.154) |
| Leukopenia | 1.439 (0.514 - 3.474) | 1.985 (0.932 - 3.996) | 1.723 (0.609 - 4.237) |
| Thrombocytopenia | 0.334 (0.018 - 1.648) | 0.886 (0.254 -2.389) |  |
| Anemia | 0.654 (0.267 - 1.452) | 1 (0.532 -1.827) | 0.763 (0.308 - 1.724) |
| ESR elevated | 0.909 (0.366 - 2.067) | 1.131 (0.58 - 2.134) | 1.478 (0.627 - 3.336) |
| low Complement | 1.45 (0.633 - 3.211) | 1.106 (0.564 - 2.101) | 1.342 (0.543 - 3.162) |
| anti-ds-DNA | 0.964 (0.446 - 2.073) | 2.387 (1.232 - 4.824) | 1.492 (0.648 - 3.555) |
| APL- Antibodies | 1.056 (0.316- 3.125) | 0.793 (0.293 - 1.945) | 1.216 (0.308 - 4.2) |
| Proteinuria | 0.628 (0.144 - 1.919) | 1.056 (0.431 - 2.331) | 0.37 (0.058 - 1.31) |
| Hematuria | 0.999 (0.324 - 2.558) | 0.987 (0.428 - 2.076) | 0.749 (0.214 - 2.037) |

ESR = erythrocyte sedimentation rate. APL = antiphospholipid. Odds ratio for thrombocytopenia in anti-B83 positive patients could not be calculated since no anti-B83 positive patient had thrombocytopenia.

Supplementary Figure 4. Graphical presentation of multivariate regression taking disease duration (diagnosis - blood sampling) and -activity as predictors and possibility of positive autoantibodies as outcome. Two activity stratified lines show the probability of having autoantibodies over the years of disease duration. Adjusted odds ratios for being autoantibody positive per one-year disease duration were (A) 0.98 (CI’s= 0.95 - 1) (B) 0.97 (CI’s= 0.95 – 0.99) and (C) 0.94 (CI’s= 0.9 – 0.98). Adjusted odds ratio for being autoantibody positive in case of active SLE disease were (A)1.43 (CI’s= 0.9 - 2.27), (B) 1.67 (CI’s = 1.03 – 2.68) and (C) 4.86, (CI’s= 2.67- 9.08).
